# Insights into sugar metabolism during bilberry ( *Vaccinium myrtillus* L.) fruit development

**DOI:** 10.1111/ppl.13657

**Published:** 2022-03-14

**Authors:** Amos Samkumar, Katja Karppinen, Binita Dhakal, Inger Martinussen, Laura Jaakola

**Affiliations:** ^1^ Department of Arctic and Marine Biology UiT The Arctic University of Norway Tromsø Norway; ^2^ Division of Food Production and Society Norwegian Institute of Bioeconomy Research Ås Norway

## Abstract

Bilberry fruit is regarded as one of the best natural sources of anthocyanins and is widely explored for its health‐beneficial compounds. Besides anthocyanins, one of the major attributes that determine the berry quality is the accumulation of sugars that provide sweetness and flavor to ripening fruit. In this study, we have identified 25 sugar metabolism‐related genes in bilberry, including invertases (INVs), hexokinases (HKs), fructokinases (FKs), sucrose synthases (SSs), sucrose phosphate synthases (SPSs), and sucrose phosphate phosphatases (SPPs). The results indicate that isoforms of the identified genes are expressed differentially during berry development, suggesting specialized functions. The highest sugar content was found in ripe berries, with fructose and glucose dominating accompanied by low sucrose amount. The related enzyme activities during berry development and ripening were further analyzed to understand the molecular mechanism of sugar accumulation. The activity of INVs in the cell wall and vacuole increased toward ripe berries. Amylase activity involved in starch metabolism was not detected in unripe berries but was found in ripe berries. Sucrose resynthesizing SS enzyme activity was detected upon early ripening and had the highest activity in ripe berries. Interestingly, our transcriptome data showed that supplemental irradiation with red and blue light triggered upregulation of several sugar metabolism‐related genes, including α‐ and β‐amylases. Also, differential expression patterns in responses to red and blue light were found across sucrose, galactose, and sugar‐alcohol metabolism. Our enzymological and transcriptional data provide new understanding of the bilberry fruit sugar metabolism having major effect on fruit quality.

## 1 INTRODUCTION

Carbohydrates are primarily formed during photosynthesis, being the main energy source for plant growth and development (Rolland et al., 2006). They are comprised of simple building units of monosaccharides (e.g., glucose, fructose, and galactose), which can be combined through glycosidic bonds to form complex molecules, such as disaccharides (sucrose), oligosaccharides (stachyose, raffinose), polysaccharides (starch), and derived sugar‐alcohols (Hu et al., 2016; Moing, 2000). Glucose, fructose, and sucrose are classified as soluble sugars. Starch is a non‐soluble sugar that accumulates in storage organs, such as plastids in leaves, roots, stems, and fruits, and can be utilized only as a reserve energy source (Cho & Kang, 2020; Wang et al., 2013). In addition to being precursors for energy‐yielding processes, the soluble sugars have been identified as signaling molecules in various plant metabolic processes during fruit ripening (Jia et al., 2013) and are also known to be involved in stress and defense responses (Tauzin & Giardina, 2014). During plant development, soluble sugars are transported from photosynthetic source tissues, such as leaves, toward sink tissues, including fruit, root, and shoot (Hammond & White, 2008). In fruits, the amount and type of sugars accumulating during the ripening improve the sweetness and flavor of fruits, thus affecting the quality of fleshy fruits (Borsani et al., 2009). In most fleshy fruits, glucose, fructose, and sucrose constitute the major proportion of sugar content, followed by trace amounts of other carbohydrates and sugar‐alcohols (Dai et al., 2016).

**FIGURE 1:**
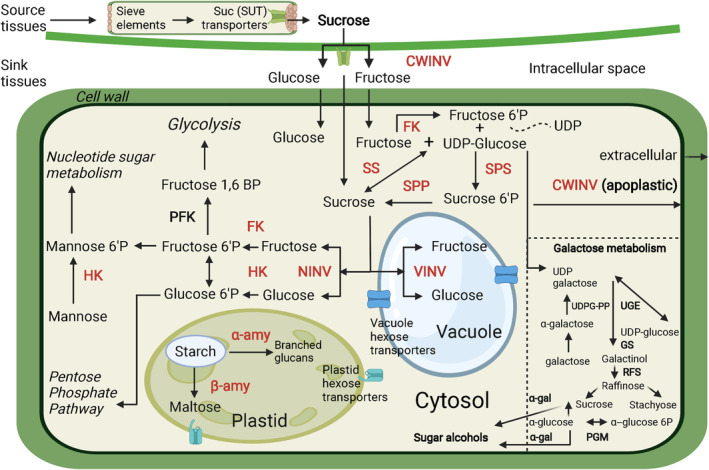
Schematic representation of sugar metabolism in fruits (modified from Chen et al., 2021). A pathway of the galactose metabolism and the key enzymes involved are also shown. The key enzymes discussed in this study are highlighted in red color. CWINV, cell wall invertase; FK, fructokinase; GS, galactinol synthase; HK, hexokinase; NINV, neutral invertase; PFK, phosphofructokinase; PGM, phosphoglucomutase; RFS, raffinose synthase; SPP, sucrose phosphate phosphatase; SPS, sucrose phosphate synthase; SS, sucrose synthase; UDPG‐PP, UDP‐galactose pyrophosphorylase; UGE, UDP‐galactose epimerase; VINV, vacuolar invertase; α‐amy, α‐amylase; α‐gal, α‐galactosyltransferase; β‐amy, β‐amylase

Carbohydrates are partitioned and transported via different transporter protein families across source and sink tissues (Julius et al., 2017). Sucrose is the major sugar transported to sink tissues via phloem during fruit development. Sucrose transporters (SUT) are mostly involved in long‐distance transport via sieve elements, whereas several organelle‐localized transporters are also involved in intercellular hexose transport (Doidy et al., 2012). In fruit tissues, sucrose is either hydrolyzed to hexoses, such as glucose and fructose, by the invertases (INVs) or converted by sucrose synthase (SS) to fructose and UDP‐glucose (Verma et al., 2011). A schematic representation of sugar metabolism in fruits is shown in Figure 1. Three types of INVs are known to be involved in sucrose hydrolysis and degradation. A neutral invertase (NINV), which is predominantly localized in the cytosol, and two acid INVs, a soluble INV localized in vacuoles (VINV) and an insoluble form found in the cell wall (CWINV), has been shown to be involved in plant sugar metabolism (Ruan et al., 2010; Wan et al., 2018). The cleaved hexoses from sucrose, that is, glucose and fructose, found in extracellular space, are further phosphorylated into glucose‐6‐phosphate (G6P) and fructose‐6‐phosphate (F6P) by hexokinase (HK) and fructokinase (FK), respectively. The hexose phosphate F6P is converted to fructose‐1,6‐bisphosphate by phosphofructokinase (PFK), which is the precursor in the energy‐yielding glycolysis process leading toward the citric acid (TCA) cycle (Granot et al., 2013). The cleaved sugar products from hydrolysis of sucrose into UDP‐glucose and F6P by SS can be further utilized in sucrose resynthesis by sucrose phosphate synthase (SPS) and sucrose phosphate phosphatase (SPP). The sucrose degradation and its resynthesis, known as “futile sucrose recycle,” is critical for the accumulation of fruit sugars and, thus, plays a key role in fruit development (Nguyen‐Quoc & Foyer, 2001).

As the primary determinants of fruit quality, sugars also vary in their composition and accumulation across fruit crops. In climacteric fruits, such as apples and tomatoes, a gradual increase of sugar content can be seen toward fruit maturation (Li et al., 2012; Nguyen‐Quoc & Foyer, 2001), whereas in non‐climacteric fruits, such as grapes and strawberries, a rapid accumulation of sugars is reported only at later stages of ripening (Akšić et al., 2019; Zhu et al., 2017). Earlier studies on sugar metabolism related to fruit ripening have mainly focused on a few genes or isoforms and are not comprehensive studies (Dai et al., 2016; Zhu et al., 2017).

Apart from flavor enhancement, the roles of sugars in fruit development are diverse: galactose, another important hexose sugar found comparatively only in very low amounts in fleshy fruits, has a major role in the reduction of cell wall loosening during fruit ripening (Althammer et al., 2020; Brummell, 2006). Unlike sucrose metabolism, the galactose pathway in plants is poorly understood. Galactose moieties are often associated with the production of raffinose‐type oligosaccharides and cell wall‐localized polysaccharides, such as stachyose, which are derived from sucrose metabolism (Gangl & Tenhaken, 2016). Furthermore, sugar alcohols, such as sorbitol, myo‐inositol, and galactinols, are synthesized by α‐galactosyltransferases (α‐gals), and they are known to be involved in protecting fruit tissues from dehydration by maintaining the cellular turgor pressure (Loescher, 1987). The hexose interconversion reactions in these pathways are usually mediated by UDP‐glucose‐pyrophosphorylase (UDPG‐PP), phosphoglucoisomerase (PGI), and phosphoglucomutase (PGM) enzymes (Decker & Kleczkowski, 2019; Figure 1). Some studies have shown a correlation between insoluble starch accumulation and soluble sugar content in ripe fruits (Cho & Kang, 2020). However, the breakdown of starch by α‐ and β‐amylase could also contribute toward a significant increase in sugar content at later stages of fruit development (Souleyre et al., 2004).

Bilberry (*Vaccinium myrtillus* L.) is an important wild berry species native to Northern Eurasian regions gaining worldwide economic importance due to a high level of anthocyanins accumulating during fruit ripening (Pires et al., 2020). In *Vaccinium* berries, the sugars mostly accumulate at the later stages of ripening upon pigmentation. The glycoside residues in bilberry anthocyanin compounds are mostly glucoside, galactoside, and arabinoside derivatives (Kähkönen et al., 2003; Zoratti et al., 2014). Earlier, we have shown that specific light wavelengths, such as red and blue light, can improve the accumulation of anthocyanin in bilberry fruit (Samkumar et al., 2021), but further information on sugar metabolism, transport and signaling is still lacking. Red and blue light wavelengths have been shown to selectively induce sugar metabolism in tomato and lettuce (Chen et al., 2019; Li et al., 2017).

The current study aims to shed light on sugar biosynthesis and metabolism in developing bilberry fruits through quantification of total and individual carbohydrate content, analyzing related gene expression, and through enzyme activity assays. This study also highlights the role of spectral light quality, specifically the effect of red and blue light, on sugar metabolism identified from our light‐treated bilberry fruit transcriptome dataset. The results provide a deeper understanding of the sugar accumulation and metabolism during fruit development in bilberries. Thus, this study adds further knowledge toward improving fruit quality and the economic value of *Vaccinium* berries and their potential marketability.

## 2 MATERIALS AND METHODS

### 2.1 Plant materials

Wild bilberry (*V. myrtillus* L.) fruits at four different developmental stages were collected from natural forest stands in Oulu (65°01′N, 25°28′E), Finland and Tromsø (69°71′N, 19°41′E), Norway. The developmental stages were small unripe green berries (S2), large unripe green berries (S3), ripening purple berries (S4), and fully ripe blue‐colored berries (S5) as previously described (Karppinen et al., 2013). The samples were kept at −80°C until used for RNA extraction and analyses of sugar content. For enzyme extraction, fresh berries were utilized. Approximately 5–15 berries were combined for one biological replicate, and at least three biological replicates were used for the analyses.

The earlier published transcriptome dataset from red and blue light treated bilberry fruits was utilized in this study (Samkumar et al., 2021). The bilberry bushes with fruits at stage S2 were collected from a natural forest stand during summer, when the daylight is almost 24 h in arctic latitudes, and kept for a few days under similar light conditions in phytotron at 16°C for acclimatization until stage S3 berries were achieved. The bushes were then placed in chambers covered from sides with photo reflective sheets and irradiated from top continuously with blue (460 nm) or red (660 nm) light wavelengths with photon fluence rate ranging between 8.0 and 10.0 μmol m^−2^ s^−1^. In addition to the specific light wavelengths, the plants received continuous ambient white light (400–700 nm) from the top. The control bilberry bushes were provided only with continuous ambient white light (400–700 nm). The temperature was maintained at 16°C. Berries were collected 6 days from the beginning of the light treatments for RNA sequencing and constructing transcriptome libraries. The S5 stage berries from the light treatments were

collected for analysis of the sugar content.

The raw reads of the transcriptome were retrieved from the BioProject ID PRJNA747684 from the NCBI‐SRA database. The top differentially expressed genes (DEGs) were analyzed and subjected to KEGG pathway enrichment analysis using the Blast2GO software suite. All the genes classified under sugar metabolic pathways were filtered out and further analyzed.

### 2.2 Identification of sugar metabolism genes

Genes encoding the major groups of sugar metabolism enzymes corresponding to CWINV, NINV, VINV, FK, HK, SS, SPP, and SPS were retrieved from transcriptome shotgun assembly (TSA) sequence databases of *Vaccinium virgatum* (TSA ID, GGAB00000000.1) and transcriptomes of *V. myrtillus* (SRA IDs, SRX3387852 and SRX3387853; Bioproject ID, PRJNA747684).

### 2.3 Phylogenetic analysis

Multiple sequence alignments of the deduced amino acid of sugar gene sequences were performed using the Clustal Omega program (https://www.ebi.ac.uk/Tools/msa/clustalo/). Phylogenetic analysis was performed for the aligned sequences to analyze the relationship between the sugar enzyme families across fruit crop species. The unrooted phylogenetic tree was constructed using the MEGA X software package (Kumar et al., 2018) using a maximum‐likelihood method with bootstrap test value set to 500 replicates in a Poisson‐distributed model.

### 2.4 RNA extraction and qRT‐PCR analysis

The frozen berries were ground to a fine powder under liquid nitrogen using mortar and pestle. Total RNA was isolated from approximately 120 mg tissue powder using Spectrum Plant Total RNA kit (Sigma‐Aldrich, St. Louis, MO, USA) following the manufacturer's instructions. The residual DNA was eliminated with on‐column digestion using DNase I (Sigma‐Aldrich). The RNA was qualified and quantified using a NanoDrop™ 2000c spectrophotometer (Thermo Fischer Scientific, Waltham, MA, USA). First‐strand cDNA was synthesized from 4 μg of total RNA by using Invitrogen Superscript IV reverse transcriptase (Thermo Fisher Scientific) according to the manufacturer's instructions.

Real‐time quantitative reverse transcription PCR (qRT‐PCR) analysis was performed with CFX96 Real‐Time System (Bio‐Rad, Hercules, CA, USA) using SsoFast™ EvaGreen supermix (Bio‐Rad) in 15 μl reaction volume. The PCR conditions were 95°C for 30 s followed by 40 cycles at 95°C for 5 s, and 60°C for 10 s. The program was followed by a melting curve analysis ranging from 65°C to 95°C with an increment of 0.5°C every cycle. All analyses were performed with three biological replicates. The results were analyzed using CFX connect software (Bio‐Rad) using 2^(−ΔΔCq)^ method. The relative expression levels were normalized with either glyceraldehyde‐3‐phosphate dehydrogenase (*GAPDH)* or *actin* with similar results. Primer sequences for genes used in this study are listed in Table S1.

### 2.5 Enzyme activity assays

A slightly modified extraction protocol previously described by Xie et al. (2009) was used for enzyme activity assays. All assays were carried out at 0–4°C, and pre‐chilled vials were used. Approximately 1 g of freshly grounded fruit tissue was extracted with 1:8 (w/v) extraction buffer containing 50 mM HEPES‐NaOH (pH 7.5), 10 mM MgCl_2_, 2.5 mM DTT, 1.0 mM EDTA, 0.05% (v/v) Triton X‐100, 0.1% (w/v) BSA, 0.1% (v/v) β‐mercaptoethanol, and 2% (w/v) polyvinylpolypyrrolidone (PVPP). The reaction mixture was centrifuged at 12,000*g* for 15 min. The crude extract was dialyzed using a cellulose membrane (molecular cut‐off 14,000 Da; Sigma‐Aldrich) for 16 h with dialysis buffer containing 25 mM HEPES‐NaOH (pH 7.5) and 0.25 mM disodium‐EDTA. The pellet was homogenized two times with 10 ml extraction buffer and then resuspended in 3 ml of 50 mM HEPES‐NaOH (pH 7.5) and 0.5 mM disodium‐EDTA. The pellet was further washed with 200 ml extraction buffer (1:40, v/v) without PVPP to assay the insoluble CWINV. The activity of VINV and NINV was measured according to Lowell et al. (1989). The reaction solution contained 80 mM K_3_PO_4_‐acetate (pH 4.5 for acidic and pH 7.5 for neutral INV) with 500 mM sucrose. The sample amount of 0.3 ml crude enzyme extract was incubated for 40 min at 37°C with reaction solution in 1 ml reaction volume. The enzymatic reaction was terminated by adding 600 μl of 1% (w/v) 3,5 dinitro salicylic acid (DNS) and further boiled for 5 min. The absorbance was read at 540 nm in a spectrophotometer (Smart Spec; Bio‐Rad). The activity of all INVs was expressed as the amount of glucose produced (mmol g^−1^ h^−1^) of fresh weight (FW).

Activities of SS and SPS were measurements according to Zhang et al. (2011). The enzyme activities were estimated similarly but by replacing the substrates; fructose for SS and F6P for SPS in the reaction mixtures. The reaction solution consisted of 0.5 M HEPES‐NaOH (pH 7.5), 0.14 M MgCl_2_, 0.028 M disodium EDTA, 0.112 M F6P or 0.084 M fructose and 0.042 M uridine diphosphate glucose (UDP‐G). The enzyme‐reaction solution mixture was incubated at 37°C for 40 min, and the reaction was stopped by adding 1.0 M NaOH. The residual F6P from the reaction mixture was further degraded by boiling the samples at 100°C for 10 min. After incubation, 0.25 ml of resorcinol solution (0.1% (w/v) in ethanol) and 0.75 ml of 35% HCl (v/v) was added into the mixture, and the samples were further incubated at 80°C for 8 min. The absorbance values were measured at 520 nm in a spectrophotometer (Smart Spec; Bio‐Rad), and the generated sucrose was expressed as the amount of sucrose generated (mmol g^−1^ h^−1^ FW).

Starch degradation activity by α‐ and β‐amylase was measured according to Hagenimana et al. (1994). The reaction mixture consisted of 0.25 ml of 100 mM phosphate buffer (pH 6.0), 0.25 ml of enzyme extract and 0.5 ml of 1% (w/v) starch solution. The mixtures were incubated for 5 min at 40°C and terminated by adding 0.4 M NaOH. The generated reducing sugars were determined by DNS reagent‐based method (Miller, 1959). For determining the α‐amylase activity, a similar assay was performed, but the enzyme crude extract was pre‐incubated at 15 min at 70°C to deactivate β‐amylase at the beginning of the reaction. The activity of β‐amylase was determined by the difference in total amylase and α‐amylase activity and expressed as the amount of maltose produced in mmol g^−1^ h^−1^ FW. All enzyme assays were performed with four biological replicates.

### 2.6 Measurement of sugar content

Part of the berry samples ground for RNA extraction were dried in a freeze dryer (Virtis benchtop‐K; SP Scientific, Gardiner, NY, USA). The dried sample powder of 0.1 g was extracted with 12 ml water containing 0.12 g PVPP in an orbital shaker for 90 min. The extracts were centrifuged at 4500*g* for 10 min, and the supernatant was filtered using a 0.2 μm filter (Merck‐Millipore, Darmstadt, Germany) and stored at −20°C until used for sugar content analysis.

Total sugar content was analyzed according to the phenol‐sulfuric acid method described by Nielsen (2010). The absorbance was read at 490 nm and quantified against a glucose standard curve with known concentrations. Individual soluble sugars were analyzed using a Sucrose/d‐Glucose/d‐Fructose assay kit (R‐Biopharm, Darmstadt, Germany). The absorbances were measured at 340 nm with a spectrophotometer (Smart Spec; Bio‐Rad). The absorbance difference for each sugar was calculated using the formula provided by the manufacturer. All sugar analyses were analyzed at least with three biological replicates.

### 2.7 Statistical analysis

The significances in gene expression, enzyme activities, and sugar levels across different berry developmental stages were analyzed using Student's *t*‐test (*P* ≤0.05). The concentrations of sugars from different light treatments were analyzed by one‐way ANOVA followed by Tukey's post‐hoc test (*P* <0.05). All the visualizations and statistical analyses were performed using Origin pro software v2021b (OriginLab Corporation, Northampton, MA, USA).

## 3 RESULTS AND DISCUSSION

### 3.1 Sugar content during bilberry fruit development

The measurement of total sugar content showed that the highest sugar levels were found in fully ripe S5 berries, followed by the S4 berries, while the lowest sugar content was found in S3 berries (Table 1). Previous studies on strawberries have shown a similar pattern of soluble sugar accumulation with an increase only at later developmental stages coinciding with the color development (Tian et al., 2012; Topcu et al., 2022; Wang et al., 2018). Although the used phenol‐sulfuric acid method measures all sugars, the total sugar amount was verified by calculating the total amount of measured individual soluble sugars (glucose, fructose, and sucrose).

**TABLE 1:** Sugar content during bilberry fruit development^a^

Ripening stage	Glucose	Fructose	Sucrose	Total sugars^b^	Total sugars^c^
S2^d^	70.06 ± 0.52^2^	78.04 ± 0.77^2^	4.57 ± 0.45^1^	152.67 ± 1.74^2^	135.63 ± 13.45^2^
S3	35.90 ± 1.54^1^	47.29 ± 2.59^1^	7.48 ± 1.09^2^	90.67 ± 5.22^1^	87.56 ± 5.45^1^
S4	101.11 ± 6.91^3^	123.69 ± 9.88^3^	7.97 ± 0.76^2^	232.77 ± 17.55^3^	214.55 ± 27.67^3^
S5	165.76 ± 8.12^4^	227.55 ± 4.34^4^	4.55 ± 0.39^1^	397.86 ± 12.85^4^	333.36 ± 27.56^4^

^a^ Data are expressed in mg g^−1^ of dry weight (DW) and represent means ± sd obtained from three biological replicates. Different superscript numbers indicate significant difference in comparison with the previous developmental stage using Student's *t*‐test (*P* ≤0.05).

^b^ Total sugar content was calculated from individual soluble sugar content quantified using Sucrose/d‐Glucose/d‐Fructose assay kit.

^c^ Total sugar content was quantified using phenol‐sulfuric acid method.

^d^ Fruit developmental stages: S2, small unripe green fruit; S3, large unripe green fruit; S4, ripening purple fruit; S5, fully ripe blue fruit.

The measurements of individual soluble sugar in bilberry fruits showed that fructose concentrations were slightly, but not significantly, higher than glucose in all developmental stages, whereas the sucrose concentration was significantly lower than the other two soluble sugars (Table 1). Also, in earlier studies, fructose was found to be the predominant sugar in ripe bilberry fruits, followed by glucose, while sucrose was found in relatively low amounts (Dare et al., 2022; Mikulic‐Petkovsek et al., 2015; Milivojević et al., 2012; Uleberg et al., 2012). Further, our results showed that the fructose and glucose levels followed a similar trend during fruit development. In small unripe green fruits (S2), fructose and glucose concentration were measured at 78 and 70 mg g^−1^ DW, respectively, before decreasing nearly two‐fold at the S3 stage and then increasing again by approximately 1.5‐fold at the S4 stage (Table 1). The fructose concentration of ripe berries (S5) was 228 mg g^−1^ DW, and glucose concentration was 166 mg g^−1^ DW. The sucrose concentration was 4.6 mg g^−1^ DW at the S2 and S5 stages but was slightly higher at the S3 and at the S4 stages of fruit development (Table 1). Our results are in accordance with a recent study, which indicated that fructose and glucose concentrations accumulate along with the anthocyanins during bilberry fruit development (Dare et al., 2022).

### 3.2 Identification and phylogenetic analysis of sugar metabolism‐related gene families in bilberry

The genes coding for members of sugar metabolism enzyme families, including CWINV, VINV, NINV, HK, FK, SPP, SPS, and SS, were retrieved from the available fruit transcriptome datasets of *Vaccinium* species (Nguyen et al., 2018; Samkumar et al., 2021). Altogether, 25 sugar metabolism pathway genes were identified; three isoforms of CWINV, two isoforms of VINV, five isoforms of NINV, four isoforms of HK, five isoforms of FK, three isoforms of SPS, two isoforms of SPP, and one isoform of SS. All the identified sugar metabolism‐related *Vaccinium* sequences, with corresponding bilberry sequences retrieved from SRA transcriptome datasets, are presented in Table S2.

**FIGURE 2:**
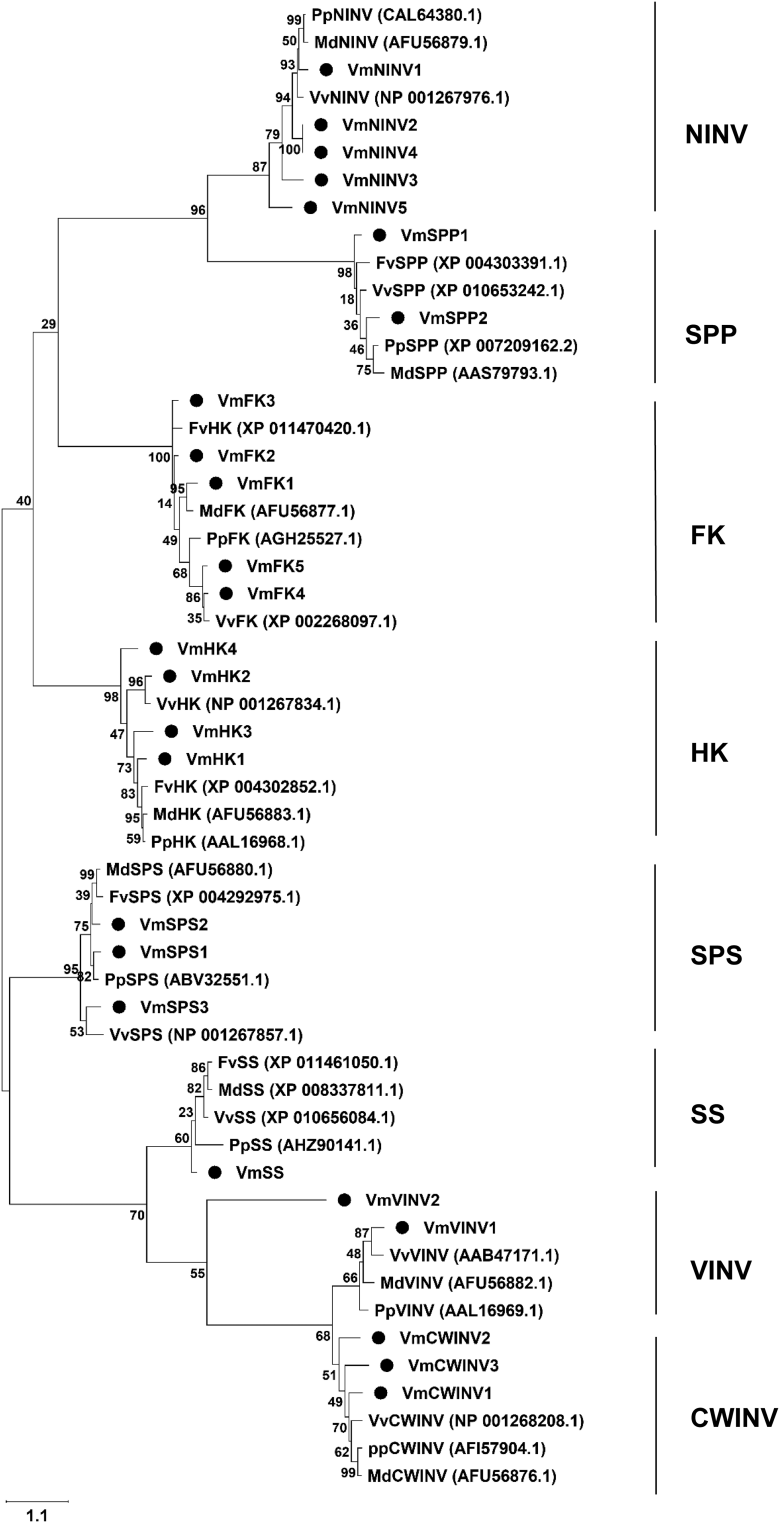
Phylogenetic analysis of bilberry sugar metabolism‐related genes. The identified gene isoforms are indicated with black circles. Numbers near each branch represents bootstrap estimates for 500 replicates. The scale bar value of 1.1 represents the amount of genetic change or nucleotide substitutions per site. CWINV, cell wall invertase; FK, fructokinase; Fv, *Fragaria vesca*; HK, hexokinase; Md, *Malus × domestica*; NINV, neutral invertase; Pp, *Prunus persica*; SPP, sucrose phosphate phosphatase; SPS, sucrose phosphate synthase; SS, sucrose synthase; VINV, vacuolar invertase; Vm, *Vaccinium myrtillus*; Vv, *Vitis vinifera*

Although it is widely believed that NINVs are only found in the cytoplasm, a previous study has shown that they can also be found in plastids (Murayama & Handa, 2007). Based on our subcellular localization analysis, three isoforms (NINV1, NINV2, and NINV4) were predicted most likely to be present in plastids rather than in the cytoplasm (Table S2). Another study showed that sucrose could enter inside plastids, but the sucrose metabolism by INVs inside the organelle is relatively unknown (Gerrits et al., 2001). The identified bilberry CWINVs and VINVs were localized in the cell wall and vacuole, respectively (Table S2). The constructed phylogenetic tree confirmed the identity of the bilberry sugar metabolism genes as well as the identity of the different INV families clustering them clearly into separate clades (Figure 2). CWINV and VINV showed a closer relationship with each other, considering that they are both acidic INVs (Roitsch & González, 2004). NINVs are branched separately due to the changes in exon numbers to that of the acidic INVs family of proteins (Duan et al., 2021). HK and FK were grouped close together because of their similar phosphorylating nature of hexoses (Granot et al., 2014), and SS and SPS were branched separately, since the SPS family of proteins has a unique S6PP domain.

### 3.3 Expression of sugar metabolism genes during bilberry fruit development

**FIGURE 3:**
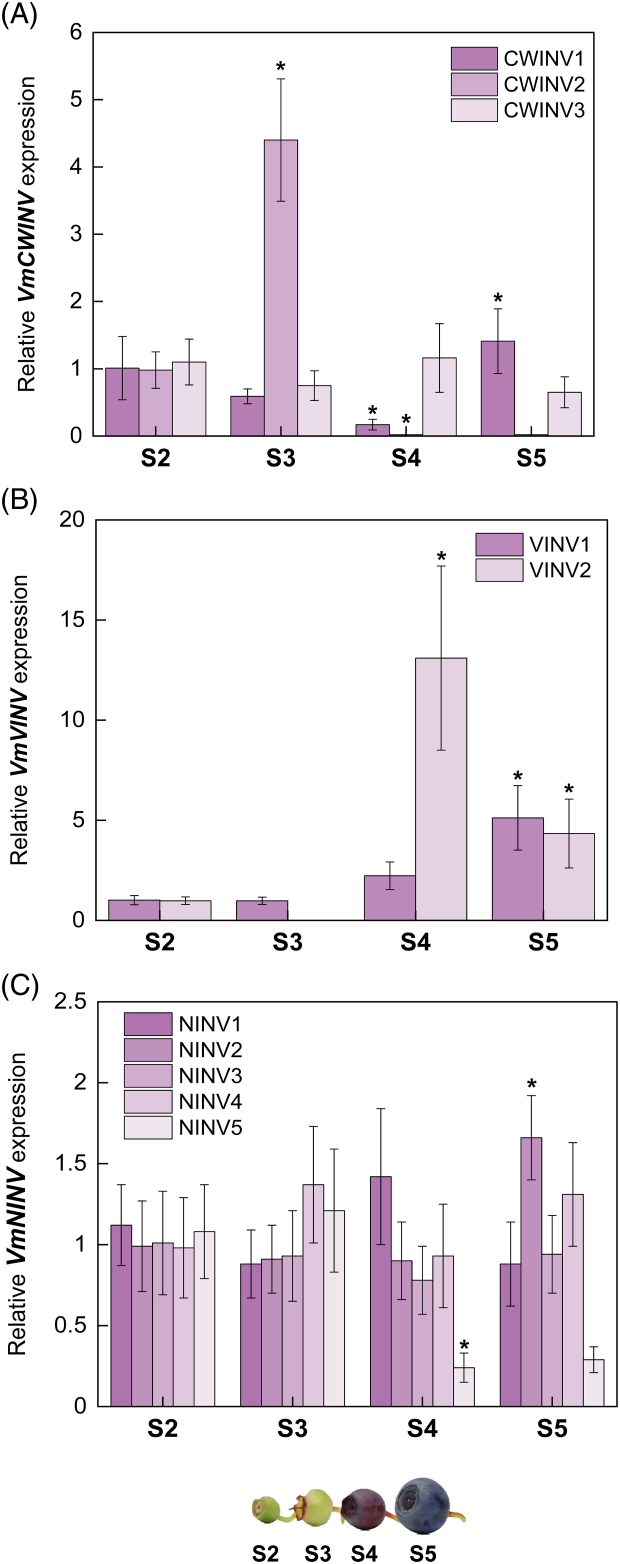
Expression of (A) *CWINV*, (B) *VINV*, and (C) *NINV* genes during bilberry fruit development. The relative gene expression levels were quantified by qRT‐PCR and normalized to *GAPDH*. The values are means of four biological replicates ± sd. Asterisks indicate significant difference in comparison with the previous developmental stage using Student's *t*‐test (*P ≤0.05). S2, small unripe green fruit; S3, large unripe green fruit; S4, ripening purple fruit; S5, fully ripe blue fruit

The relative gene expression levels of all the identified gene isoforms were determined during berry development by using qRT‐PCR. The expression patterns of all three isoforms of *CWINV* were different from one another (Figure 3A). *CWNV1* expression was low in S3 and S4 fruits but slightly higher in the ripe fruit stage (S5), whereas *CWINV2* showed a significant four‐fold higher expression in S3 than in the S2. In the later stages of ripening (S4 and S5), there was no *CWINV2* expression detected. The expression of *CWINV3* did not vary significantly across the fruit development and ripening stages, being slightly lower in S3 than in S2, whereas it showed similar expression in S4 and again slightly decreased its expression in fully ripe fruit (Figure 3A). CWINVs are vital during sink organ development, such as fruit, and play a major role in fruit setting (Ruan et al., 2010), which might explain why the S3 stage in bilberries is critical during fruit development evidenced from *CWINV2* of the isoforms (Figure 3A). Generally, CWINVs are the key enzymes involved in partitioning to hexoses once the sucrose is transported from source tissues and upregulate the sink strength in fruits (Roitsch & González, 2004). From our results, we can infer that all the differently expressed isoforms of *CWINV* might have distinct functions at certain developmental stages. Especially *CWINV1* might have a role in late ripening stages in increasing the levels of glucose and fructose in ripe berries, as shown in our study (Table 1).

Also, the expression of both *VINVs* showed a significant increment toward berry ripening (Figure 3B). *VINV1* had the highest expression in the S5 stage, while *VINV2* showed a 13‐fold higher expression in ripening purple fruit (S4) than the previous two unripe green fruit stages, then decreasing in fully ripe blue fruit (Figure 3B). Studies have shown that VINVs are the key determinants of storage and resynthesis of sucrose in vacuoles during mature stages of fruit development (Husain et al., 2001; Tang et al., 1999). Thus, we can speculate that the increase in expression levels of these two isoforms of *VINV* at the ripening stages S4 and S5 could be responsible for converting the accumulated sucrose in vacuoles to hexoses, and thus elevating the total sugar concentrations in fully ripe bilberry fruits (Table 1).

All the identified isoforms of *NINV* were expressed relatively stable levels during fruit ripening, except *NINV2* increasing significantly at S5 and *NINV5* decreasing significantly after S3 (Figure 3C). *NINV3* and *NINV4* showed slightly decreased expression in S4 fruit than in S3 fruit but not significant. *NINV1* had the highest expression in ripening red‐colored fruit (S4), and *NINV2* had the highest expression in blue‐colored S5 fruit (Figure 3C). A direct correlation of NINVs activity to the fructose‐to‐glucose ratios in fruits has earlier been shown by Desnoues et al. (2014). Since our results show that *NINVs* were expressed in all the developmental stages during bilberry fruit development, this could imply that stable *NINV* expression leads to the detected increase in fructose amounts (Table 1) in all developmental stages achieved by the segregation of sucrose in the cytoplasm (Hao‐Ran et al., 2017).

**FIGURE 4:**
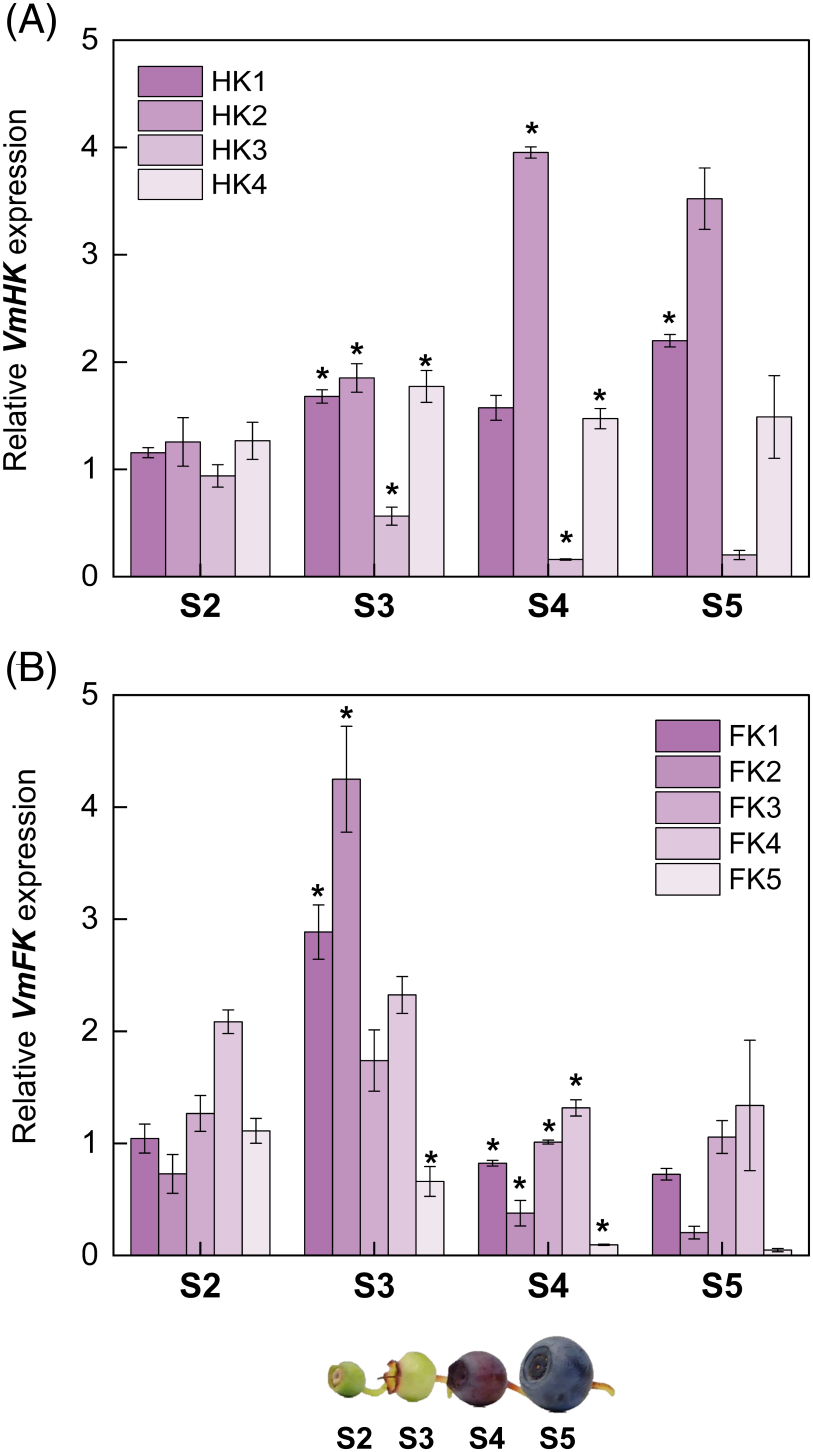
Expression of (A) *HK* and (B) *FK* genes during bilberry fruit development. The relative gene expression levels were quantified by qRT‐PCR and normalized to *GAPDH*. The values are means of four biological replicates ± sd. Asterisks indicate significant difference in comparison with the previous developmental stage using Student's *t*‐test (*P ≤0.05). S2, small unripe green fruit; S3, large unripe green fruit; S4, ripening purple fruit; S5, fully ripe blue fruit

Three isoforms of HK (*HK1*, *HK2*, and *HK4*) were similarly expressed between developmental stages S2 and S3 (Figure 4A), whereas *HK3* expression tended to decrease as the fruit ripening progressed. Afterwards, the expression of *HK2* increased significantly and to some extent by *HK1* in S4 and S5, unlike other isoforms. Glucose can only be phosphorylated by HKs, while fructose is phosphorylated by both HKs and FKs, although the affinity toward fructose moieties is higher in FKs (Granot et al., 2013). In our study, all the *FKs*, except *FK5*, were found to increase in expression after the S2 stage, and the highest expression level was found in S3 berries. However, during late developmental stages (S4 and S5), the expression levels decreased (Figure 4B). The *HKs* and *FKs* showed an interesting opposite expression patterns in early and late berry developmental stages. The result suggests that an increase in fructose content can be attributed to the interplay of FKs in the onset of ripening and by both FKs and HKs at late berry developmental stages.

**FIGURE 5:**
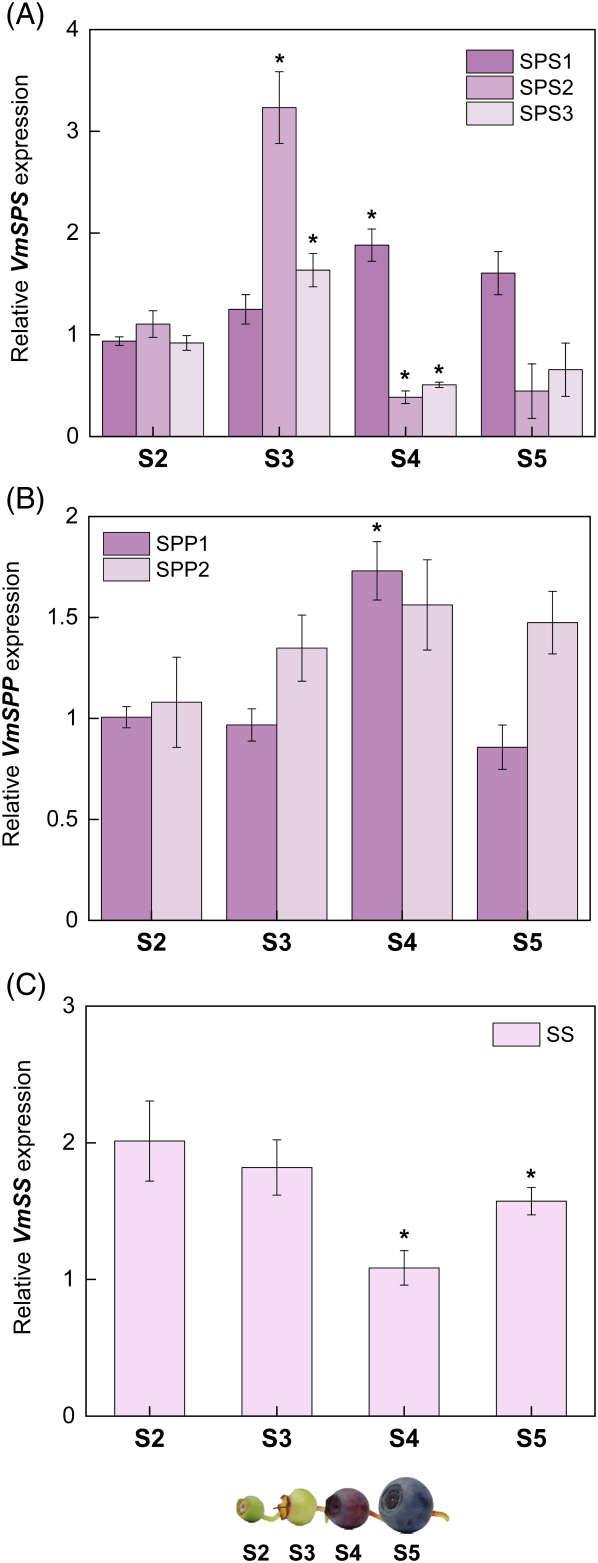
Expression of sucrose metabolism genes (A) *SPS*, (B) *SPP*, and (C) *SS* during bilberry fruit development. The relative gene expression levels were quantified by qRT‐PCR and normalized to *GAPDH*. The values are means of four biological replicates ± sd. Asterisks indicate significant difference in comparison with the previous developmental stage using Student's *t*‐test (*P ≤0.05). S2, small unripe green fruit; S3, large unripe green fruit; S4, ripening purple fruit; S5, fully ripe blue fruit

Regarding the sucrose metabolism enzymes, the expression of *SPS1* increased in late stages, whereas *SPS2* and *SPS3* expression in S4 and S5 stages were low (Figure 5A). *SPS2* expression was highest in S3 berries. SPS is the key enzyme involved in sucrose resynthesis, and an increase in expression at the onset of ripening implies that sucrose is recycled actively, especially at the S3 stage. Specifically, the expression of SPS1 might contribute to the increase in sucrose content (Table 1), in accordance with previous studies (Vimolmangkang et al., 2016). *SPPs* did not show significant variation across developmental stages, except at S4, where *SPP1* and *SPP2* levels slightly increased (Figure 5B). The only characterized SS isoform expression was higher in early berry development and tended to decrease at later stages (Figure 5C). Earlier, a similar expression trend was observed in kiwifruit ripening, where SS expression was shown to be involved in post‐sucrose unloading pathways (Chen et al., 2017). Generally, an increase in SS expression during ripening results in low sucrose levels, which has been demonstrated in two non‐climacteric fruits, grape and strawberry, in a previous study and in concordance with our findings (Tian et al., 2012).

### 3.4 Sugar‐related enzyme activities across bilberry fruit developmental stages

**FIGURE 6:**
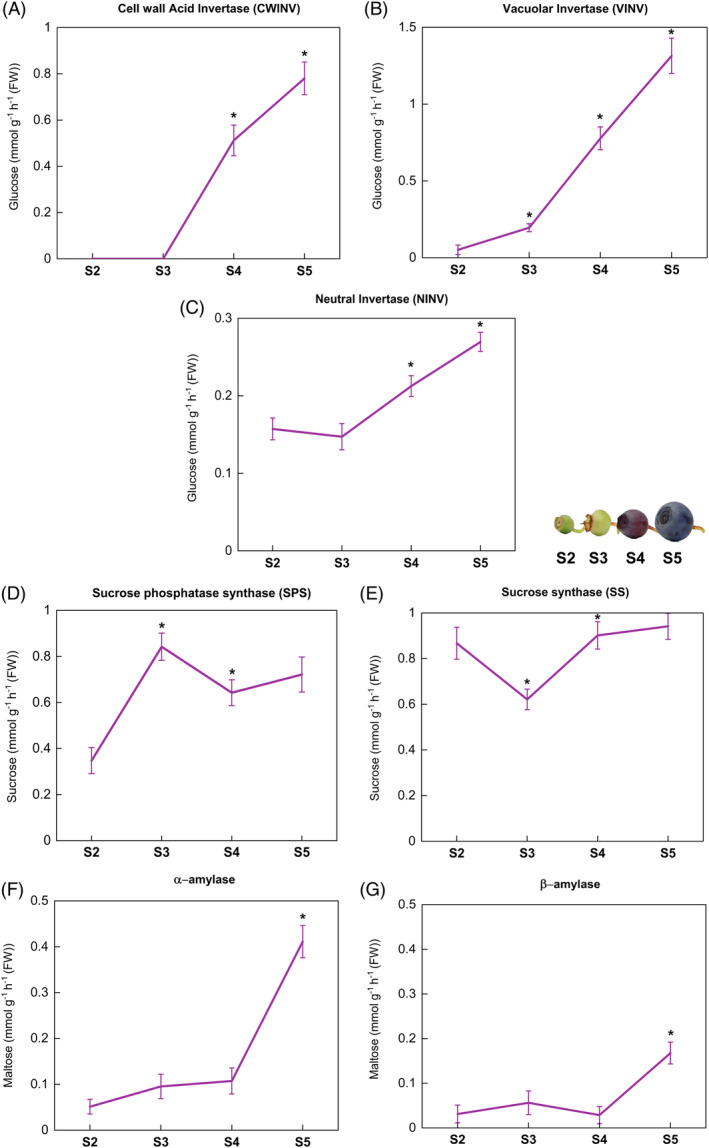
Changes in the (A) CWINV, (B) VINV, (C) NINV, (D) SPS, (E) SS, (F) α‐amylase, and (G) β‐amylase enzyme activities during bilberry fruit development. The enzyme activities are expressed in amounts of corresponding sugar released in mmol g^−1^ h^−1^ FW from each assay. The values represent mean ± sd of four biological replicates and asterisks indicate significant difference in comparison with the previous developmental stage using Student's *t*‐test (*P ≤0.05). S2, small unripe green fruit; S3, large unripe green fruit; S4, ripening purple fruit; S5, fully ripe blue fruit

Enzyme activities of CWINV and VINV were detected only in very low levels in early developmental stages (S2 and S3), but their activities increased significantly from S3 onwards, with the highest activity found in S5 berries (Figure 6A,B). Generally, CWINVs are considered sink‐specific enzymes involved in sucrose unloading at early stages in berry development (Wang et al., 2014). However, some studies have shown that gene expression and enzyme activities of CWINVs are lower in fruits and may not be directly related to apoplastic sucrose unloading at the beginning of ripening (Li et al., 2012). Contrastingly, NINV activity was found early in S2 berries, followed by a significant decrease in activity at fully ripe S5 berries (Figure 6C). Activities of both sucrose metabolism enzymes, SPS and SS, were also detected in unripe berries, but their activities tended to increase only at later stages (Figure 6D,E). These enzymes exhibit an opposite trend at the S3 stage, i.e., SPS activity was highest in S3 whereas SS activity was lowest at the same stage, which might be the critical stage in sucrose resynthesis occurring in bilberry fruits (futile cycle). Our results are consistent with earlier studies, as it has been previously demonstrated that SS activities are often higher than NINVs since the former produces reversible conversion and better homeostasis in sink tissues (Moscatello et al., 2011). SS is also likely to be involved in starch accumulation in plastids at the beginning of fruit ripening (Ross et al., 1994). However, starch degradation occurs during late berry development in plastids (Zhu et al., 2017). The activities of both the starch degrading amylases (α‐ and β‐amylase), were found to be increased toward the fully ripe berries (S5), and detected only in low levels in other stages of bilberry fruit development (Figure 6F,G). This might suggest that the stored starch granules are rapidly utilized for hexose conversion inside plastids only after the fruit has fully developed. To correlate and summarize both the gene expression and enzyme activities during the four developmental stages in bilberry fruit, we infer that both the acid INVs are involved in developing sink strength during the S3‐S4 stages. Likewise, HKs and FKs are also vital in these stages to provide the carbon source in developing berries. The major enzymes involved in soluble sugar accumulation might be SS and SPS (*SPS1*), which utilize the sucrose at the onset of ripening, and resynthesis occurs at the late ripening stages of bilberries. It also explains why the formation of UDP‐sugars by SS during S3‐S4 stages could be utilized for the production of glycosylated anthocyanins (Tian et al., 2012), which develops the color on the skin and flesh of bilberry fruits from the S4 stage onwards.

### 3.5 Effect of light spectral quality in bilberry fruit sugar metabolism

**FIGURE 7:**
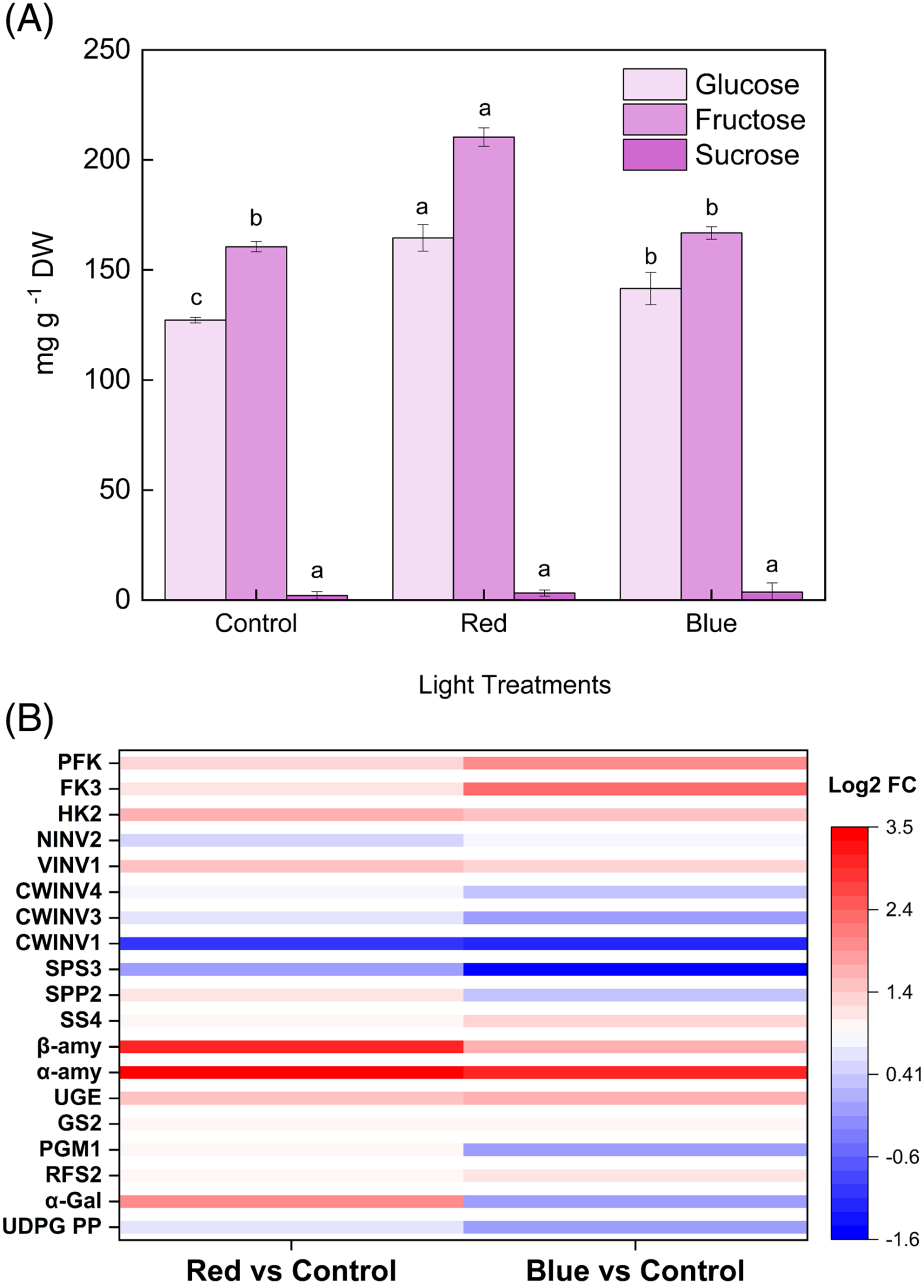
Effect of spectral light qualities to sugar metabolism in bilberry fruits. (A) Sugar content in light‐treated bilberry fruits. The values (mg g^−1^ DW) represent mean ± sd of three biological replicates. Different letters denote significant differences among light treatments analyzed by one‐way ANOVA followed by Tukey's post hoc test (*P* <0.05). (B) DEGs related to sugar metabolism in response to red and blue light. The expression levels are represented in color code boxes based on log2 fold changes. CWINV, cell wall invertase; FK, fructokinase; GS, galactinol synthase; HK, hexokinase; NINV, neutral invertase; PFK, phosphofructokinase; PGM, phosphoglucomutase; RFS, raffinol synthase; SPP, sucrose phosphate phosphatase; SPS, sucrose phosphate synthase; SS, sucrose synthase; UDPG PP, UDP glucose/galactose pyrophosphorylase; UGE, UDP‐galacturonate 4‐epimerase; VINV, vacuolar invertase; α‐amy, α‐amylase; α‐gal, α‐galactosidase; β‐amy, β‐amylase

The supplemental light quality treatment had a significant effect on bilberry fruit sugar metabolism. The individual sugar concentrations of glucose and fructose were significantly higher under red light treatment (165 and 210 mg g^−1^ DW, respectively) compared with control berries (Figure 7A). Blue light also slightly increased glucose and fructose content in ripe berries (141 and 166 mg g^−1^ DW, respectively). Sucrose levels were found to be very low under the light treatments (2–3.5 mg g^−1^ DW), and they were not found to be significantly affected by the supplemental light treatments (Figure 7A).

The top DEGs found between the control and light treatments (red, blue) are shown in Figure 7B. Both light treatments upregulated α‐ and β‐amylases, which are the key genes involved in starch degradation in plastids. Red light upregulated β‐amylase up to three‐folds, which indicates that maltose levels have been elevated inside plastids and could subsequently be converted to other hexoses, such as glucose, adding sweetness and flavor to the fully ripe fruit (Xiao et al., 2018). Blue light downregulated the genes encoding hexose inter‐conversion enzymes involved in galactose metabolisms, such as PGM, α‐galactosyltransferase (α‐gal), and UDP‐glycopyrophophorylase (UDPG‐PP) (Figure 7B). On the other hand, red light upregulated α‐galactosyltransferase gene, which is likely to be involved in producing galactose‐derived and cell wall‐bound polysaccharides (Edwards et al., 1999). Other genes of key enzymes arising from the galactose metabolism, such as galactinol synthase (GS), UDP‐galactose epimerase (UGE), and raffinose synthase (RFS), were also upregulated in red light treatment (Figure 7B). Comparatively, blue light upregulated genes encoding hexose phosphorylating enzymes, including HK, FK, and PFK, more than red light. *VINV* was upregulated by both light treatments (Figure 7B), which suggests that the degradation of sucrose to hexoses by INVs was mostly occurring in vacuolar spaces by VINVs than in the cytosol by the NINVs (Rabot et al., 2014) in response to light quality. Likewise, it is also evidenced by the *NINVs*, which show low expression under light treatments. Genes encoding sucrose enzymes (SS and SPP) were upregulated under red light, whereas SPP and SPS were downregulated under blue light (Figure 7B). All the *CWINVs* were downregulated under both light treatments. The result agrees with the earlier studies, which have shown that CWINV activities are reduced under abiotic stress during fruit set and are the most affected during altered circadian rhythm (Liu et al., 2016; Proels & Hückelhoven, 2014). For instance, UV light stress is causing downregulation of CWINV as part of the plant's response to elevated innate antioxidant mechanisms (Nishanth et al., 2018). Hence, the effect of light quality on CWINV could be a pleiotropic effect. Thus, we conclude that the light quality might be inhibiting carbohydrate utilization from source tissues intracellularly via CWINV, but C‐equivalents are utilized from hexose interconversions intercellularly from vacuoles with also an enhanced starch conversion to maltose pools from plastids, thus increasing soluble sugar concentrations and sink strength in berry tissues.

## 4 CONCLUSIONS

In the current study, we have identified and analyzed the sugar metabolism encoding genes in bilberry across the fruit developmental stages. The results showed that the gene expression and enzyme activities of acid INVs were low at the beginning of bilberry fruit development, whereas a significant increase in the expression of these irreversible sucrose‐conversion enzyme‐coding genes were detected at later ripening stages, probably responsible for glucose and fructose accumulation to ripe berries. Our results also indicate that SS and SPS are most likely the key enzymes involved in the reversible sucrose conversions, as both activity and the gene expression level (SS, SPS1) of these enzymes were consistent across all the four bilberry fruit developmental stages. Furthermore, our results indicate that all the INVs, HKs, and sucrose resynthesizing enzymes (SS, SPS) contributed to the final accumulation of sugars in fully ripe berries, where fructose and glucose were found to be the most abundant sugars. In response to light spectral quality, our transcriptomics analysis indicates that both red and blue supplemental light irradiation triggers the expression of many sugar‐related genes, including genes encoding starch degrading enzymes, which likely contribute to the increase in hexose content. In addition, both light qualities had a negative impact on CWINV expression, but the upregulation in both HKs and VINVs were likely to be responsible for the increase in glucose and fructose content under red light. Overall, the supplemental red light had a significant impact on elevating the total sugar concentrations in bilberry fruit. This study gives the first comprehensive report on bilberry fruit sugar metabolism and provides an ideal platform for further functional genomics studies on improving fruit quality.

## CONFLICT OF INTEREST

The authors declare that they have no conflicts and competing interests.

## AUTHOR CONTRIBUTIONS

Laura Jaakola and Katja Karppinen conceptualized the project. Amos Samkumar performed the enzyme activity assays, analyzed the transcriptome data, and wrote the manuscript. Binita Dhakal and Katja Karppinen performed the gene expression analyses. Katja Karppinen with the contribution of Binita Dhakal quantified the sugar content. Laura Jaakola, Katja Karppinen, and Inger Martinussen contributed to the editing and proofreading of the manuscript draft. All authors have read and approved the final manuscript.

## Supporting information


**Table S1.** List of primers used in qRT‐PCR analysis.
**Table S2.** The identified sugar metabolism genes from *V. myrtillus* fruit. The corresponding sequences are retrieved from *V. virgatum* and their TSA IDs are provided in the following column.

## Data Availability

The data that support the findings of this study are openly available in NCBI‐SRA database at http://www.ncbi.nlm.nih.gov/bioproject/747684, reference number (PRJNA747684).
