# Supplementary material for: Insights into sugar metabolism during bilberry ( Vaccinium myrtillus L.) fruit development
Source: Physiol Plant. 2022 Mar 14;174(2):e13657. doi: 10.1111/ppl.13657 (PMC9313557; doi:10.1111/ppl.13657)
Supplement: Supplementary file 1 — Table S1. List of primers used in qRT‐PCR analysis. Table S2. The identified sugar metabolism genes from V. myrtillus fruit. The corresponding sequences are retrieved from V. virgatum and their TSA IDs are provided in the following column. [file PPL-174-0-s001.docx]

**Insights into sugar metabolism during bilberry (*Vaccinium myrtillus* L*.*) fruit development**

**Amos Samkumar^1^, Katja Karppinen^1^, Binita Dhakal^1^, Inger Martinussen^2^, Laura Jaakola^1,2^**

^1^Department of Arctic and Marine Biology, UiT The Arctic University of Norway, Tromsø, Norway

^2^Norwegian Institute of Bioeconomy Research, Ås, Norway

**Supplementary information**

**Number of tables: 2**

**Supplementary Table 1.** List of primers used in qRT-PCR analysis.

| **Gene** | **Forward primer sequence** | **Reverse primer sequence** |
| --- | --- | --- |
| *VmCWINV1* | GTGGACCGAAGCCAAACAACCT | GCCCAGTCTTCGATGTCTCGAT |
| *VmCWINV2* | CTCTGATGCAACCAGTTCCTCT | CTCCTGAGGGAAAGCTTCTTGT |
| *VmCWINV3* | GGCTTCAGTCGTTTCCAAGMC | CCACCCTTGAGCTCTGTATCAT |
| *VmVINV1* | TGATGACCTCTTGAAGGGTTGG | AGCAGGGAATTCAGTACCGTTC |
| *VmVINV2* | TTGCTCAAGGAGGGAGAACAGT | TATAAGCCGAGTCCATCGACCA |
| *VmNINV1* | GGCCTGCATTAAGATGAAGAGG | TTCCCTACAAATCTCCCACTGC |
| *VmNINV2* | GAAGTCCTAGACCCAGATTTCG | CCAGTTACCTTCCCATAAGCAC |
| *VmNINV3* | GAAGGCGAGGAGTGGAGAATTA | TGCGTCTCTCGGCTATCTTAAC |
| *VmNINV4* | CCGTTAGACAGTTGGCCTGAAT | TCCTCCCAGTACAGCAAAGAAG |
| *VmNINV5* | GAGTGGTTAAGCGTCTTCATGC | TCCATTCAGGGAGAGAATCAGG |
| *VmFK1* | GCACAACTAGCTGAGGATGACA | ATTGCACCTCTCTCCTTGACAG |
| *VmFK2* | CTGAAGGGTCAGATGGTTGCAGA | AATGCATCACCAGCACCAGTCG |
| *VmFK3* | TTTACCAGGACGAGGAGCGATT | CTTTCGTCGGTAAGGCAGGAAT |
| *VmFK4* | ACCGCTGATGAACTCAATCTCG | ACCTCCATCGCCTTTAAGTGAG |
| *VmFK5* | CCAGGCTGAAGTGATAAAGGTG | CACCAATGGTCACAAGGAGAAG |
| *VmHK1* | GGAGGCAGATACTACGACTCAA | AGGTAGTAGACCATGCCACTTG |
| *VmHK2* | GGGCACTCTCGTTAAATGGACA | CACACGCATATCGAGACCAACT |
| *VmHK3* | TGTCTGCCCTGGTCAATGAT | TACTCTGGCATCCATGTCC |
| *VmHK4* | GAAGTTGAGAAGAACGGTCGTG | GATTTCAGTCACGGCTTCATCC |
| *VmSPS1* | GGCATGGTCAAAGGGTGTTCTA | CTCCTCAACGCTGGGAATTTAC |
| *VmSPS2* | AGGAGGCTTTAATCCTGTGGAC | CGGAAGACTCACCTTCTGAGTT |
| *VmSPS3* | GAGCTGCTTAGAACAGCTGGAA | CTTCGCTTCTGCATTGGTGTAG |
| *VmSPP1* | AGCACTAAGTCTTCCGTCCTGT | TGACTTGATCTACCCACACTCG |
| *VmSPP2* | GTTTGACAAGTGGGAGCTGTCT | ATACGAGCCCATCTGGTACTTC |
| *VmSS* | CGGGGTCTCTGGTTTTCACAT | CCCGTACAAAGATCCCATGTTC |
| *VmGAPDH* | CAAACTGTCTTGCCCCACTT | CAGGCAACACCTTACCAACA |
| *VmActin* | TTCCCTGGGATTGCTGATAG | GGTCTTGGCAATCCACATCT |

**Supplementary Table 2.** The identified sugar metabolism genes from *V. myrtillus* fruit. The corresponding sequences are retrieved from *V. virgatum* and their TSA IDs are provided in the following column.

| Gene name | Sequence_ID from TSA database (*V. virgatum*) | SRA_ID (*V. myrtillus*)  and identity match (%)^1^ | Unigene_ID (*V. myrtillus)*  and identity match (%)^2^ | Related species identity match (%) | Sub-Cellular locali-zation^3^ |
| --- | --- | --- | --- | --- | --- |
| *CWINV1* | [GGAB01072872.1](https://www.ncbi.nlm.nih.gov/nuccore/GGAB01072872.1) | [729392](https://trace.ncbi.nlm.nih.gov/Traces/sra/sra.cgi?run=SRR6286471.729392.1&RID=XNS2F4KS01R&display=reads) (97%) | TRINITY_DN2977_c2_g1_i2 (98%) | *NC034011 (P. persica)*  (66%) | Cell wall (0.7) |
| *CWINV2* | [GGAE01109889.1](https://www.ncbi.nlm.nih.gov/nuccore/GGAE01109889.1) | [976747](https://trace.ncbi.nlm.nih.gov/Traces/sra/sra.cgi?run=SRR6286471.976747.1&RID=XNSBKDH201R&display=reads) (97%) | TRINITY_DN58605_c0_g1_i1 (98%) | *NW021025375 (C. sinensis (82%)* | Cell wall (0.9) |
| *CWINV3* | [GGAE01121540.1](https://www.ncbi.nlm.nih.gov/nuccore/GGAE01121540.1) | [1364501](https://trace.ncbi.nlm.nih.gov/Traces/sra/sra.cgi?run=SRR6286470.1364501.1&RID=XNSGVETZ01R&display=reads) (99%) | TRINITY_DN17376_c0_g1_i10 (96%) | *NC012010 (V. vinifera)*  (64%) | Cell wall (0.3) |
| *NINV1* | [GGAE01022874.1](https://www.ncbi.nlm.nih.gov/nuccore/GGAE01022874.1) | [1523998](https://trace.ncbi.nlm.nih.gov/Traces/sra/sra.cgi?run=SRR6286471.1523998.1&RID=XNT0C6GE01R&display=reads) (99%) | TRINITY_DN2901_c0_g1_i1 (99%) | *KF718860 (C. sinensis)*  (84%) | Plastid (0.5) |
| *NINV2* | [GGAE01119103.1](https://www.ncbi.nlm.nih.gov/nuccore/GGAE01119103.1) | [783733](https://trace.ncbi.nlm.nih.gov/Traces/sra/sra.cgi?run=SRR6286471.783733.1&RID=XNT3WH7V01R&display=reads) (95%) | TRINITY_DN7461_c0_g1_i1 (93%) | *CM014052 (M. domestica)*  (76%) | Plastid (0.6) |
| *NINV3* | [GGAE01011826.1](https://www.ncbi.nlm.nih.gov/nuccore/GGAE01011826.1) | [1386518](https://trace.ncbi.nlm.nih.gov/Traces/sra/sra.cgi?run=SRR6286470.1386518.1&RID=XNT7CEZY01R&display=reads) (99%) | TRINITY_GG_15949_c0_g1_i1 (99%) | *NC_012012 (V. vinifera)*  (79%) | Cytoplasm (0.2) |
| *NINV4* | [GGAE01062287.1](https://www.ncbi.nlm.nih.gov/nuccore/GGAE01062287.1) | [1278054](https://trace.ncbi.nlm.nih.gov/Traces/sra/sra.cgi?run=SRR6286470.1278054.1&RID=XNTAK9SB01R&display=reads) (98% | TRINITY_DN141831_c0_g1_i1 (100%) | *KP053405 (C. sinensis)*  (88%) | Plastid (0.6) |
| *NINV5* | [GGAE01030565](https://www.ncbi.nlm.nih.gov/nuccore/GGAE01030565) | [1057615](https://trace.ncbi.nlm.nih.gov/Traces/sra/sra.cgi?run=SRR6286470.1057615.1&RID=XNTE3KDG01R&display=reads) (99%) | TRINITY_GG_11522_c76_g1_i1 (90%) | *NC034016 (P. persica)*  (86%) | Cytoplasm (0.2) |
| *VINV1* | [GGAE01006295.1](https://www.ncbi.nlm.nih.gov/nuccore/GGAE01006295.1) | [505506](https://trace.ncbi.nlm.nih.gov/Traces/sra/sra.cgi?run=SRR6286470.505506.1&RID=XNSPN47N01R&display=reads) (97%) | TRINITY_DN4663_c0_g1_i3 (78%) | *KU884473 (C. sinensis)*  (76%) | Vacuole (0.7) |
| *VINV2* | [GGAB01063390.1](https://www.ncbi.nlm.nih.gov/nuccore/GGAB01063390.1) | [1675652](https://trace.ncbi.nlm.nih.gov/Traces/sra/sra.cgi?run=SRR6286470.1675652.1&RID=XNSV3W6701R&display=reads) (98%) | TRINITY_DN4663_c0_g1_i3 (97%) | *NC012022.3 (V. vinifera)*  (67%) | Vacuole (0.7) |
| *HK1* | [GGAE01107266.1](https://www.ncbi.nlm.nih.gov/nuccore/GGAE01107266.1) | [1301214](https://trace.ncbi.nlm.nih.gov/Traces/sra/sra.cgi?run=SRR6286470.1301214.1&RID=4UZBK1H2014&display=reads) (100%) | TRINITY_DN381_c0_g3_i3 (99%) | *NC034015 (P. persica)*  (83%) | NA |
| *HK2* | [GGAE01118471.1](https://www.ncbi.nlm.nih.gov/nuccore/GGAE01118471.1) | [1529550](https://trace.ncbi.nlm.nih.gov/Traces/sra/sra.cgi?run=SRR6286470.1529550.1&RID=42TZM1KX014&display=reads) (99%) | TRINITY_DN381_c0_g3_i3 (85%) | *AM456450.2 (V. vinifera)*  (83%) | NA |
| *HK3* | [GGAE01031242.1](https://www.ncbi.nlm.nih.gov/nuccore/GGAE01031242.1) | - | TRINITY_DN6805_c0_g2_i7 (99%) | *JN118545.1 (V. vinifera)*  (82%) | NA |
| *HK4* | [GGAB01088020.1](https://www.ncbi.nlm.nih.gov/nuccore/GGAB01088020.1) | [1137583](https://trace.ncbi.nlm.nih.gov/Traces/sra/sra.cgi?run=SRR6286470.1137583.1&RID=42VR0VFZ014&display=reads) (99%) | TRINITY_DN6900_c1_g1_i3 (99%) | *NC041799 (M. domestica)*  (72%) | NA |
| *FK1* | [GGAE01074415.1](https://www.ncbi.nlm.nih.gov/nuccore/GGAE01074415.1) | [1269317](https://trace.ncbi.nlm.nih.gov/Traces/sra/sra.cgi?run=SRR6286470.1269317.1&RID=42XU1YXP016&display=reads) (100%) | TRINITY_GG_25277_c0_g1_i1 (98%) | *NW021026114 (C. sinensis)*  (78%) | NA |
| *FK2* | [GGAB01032707.1](https://www.ncbi.nlm.nih.gov/nuccore/GGAB01032707.1) | [1052446](https://trace.ncbi.nlm.nih.gov/Traces/sra/sra.cgi?run=SRR6286471.1052446.1&RID=4XDCCKPH014&display=reads) (98%) | TRINITY_DN1834_c1_g3_i2 (99%) | *XM008378279 (M. domestica)*  (84%) | NA |
| *FK3* | [GGAB01048669.1](https://www.ncbi.nlm.nih.gov/nuccore/GGAB01048669.1) | [232383](https://trace.ncbi.nlm.nih.gov/Traces/sra/sra.cgi?run=SRR6286470.232383.1&RID=42Z38UKK016&display=reads) (97%) | TRINITY_DN28664_c0_g2_i3 (99%) | *JX067537 (A. chinensis)*  (85%) | NA |
| *FK4* | [GGAB01032403.1](https://www.ncbi.nlm.nih.gov/nuccore/GGAB01032403.1) | [1177935](https://trace.ncbi.nlm.nih.gov/Traces/sra/sra.cgi?run=SRR6286470.1177935.1&RID=4XJRY898016&display=reads) (99%) | TRINITY_GG_8267_c0_g1_i1 (98%) | *JX067535 (A. chinensis)*  (90%) | NA |
| *FK5* | [GGAB01084714.1](https://www.ncbi.nlm.nih.gov/nuccore/GGAB01084714.1) | [1423238](https://trace.ncbi.nlm.nih.gov/Traces/sra/sra.cgi?run=SRR6286471.1423238.1&RID=4XMXVBFZ016&display=reads) (96%) | TRINITY_GG_983_c0_g1_i1 (97%) | *NC012011 (V. vinifera)*  (86%) | NA |
| *SS* | [GGAE01053339](https://www.ncbi.nlm.nih.gov/nuccore/GGAE01053339) | [826437](https://trace.ncbi.nlm.nih.gov/Traces/sra/sra.cgi?run=SRR6286470.826437.1&RID=504RZ3TG014&display=reads) (97%) | TRINITY_GG_1104_c141_g1_i1 (99%) | *NC041805 (M. domestica)*  (77%) | NA |
| *SPP1* | [GGAE01112488.1](https://www.ncbi.nlm.nih.gov/nuccore/GGAE01112488.1) | [664663](https://trace.ncbi.nlm.nih.gov/Traces/sra/sra.cgi?run=SRR6286470.664663.1&RID=4XVUWGSP016&display=reads) (99%) | TRINITY_DN6169_c0_g1_i1 (99%) | *AY509994 (A. chinensis)*  (87%) | NA |
| *SPP2* | [GGAB01063835.1](https://www.ncbi.nlm.nih.gov/nuccore/GGAB01063835.1) | [1470994](https://trace.ncbi.nlm.nih.gov/Traces/sra/sra.cgi?run=SRR6286471.1470994.1&RID=4XWMAT00016&display=reads) (96%) | TRINITY_DN43187_c0_g1_i1 (98%) | *NC012014.3 (V. vinifera)*  (68%) | NA |
| *SPS1* | [GGAE01109516.1](https://www.ncbi.nlm.nih.gov/nuccore/GGAE01109516.1) | [554516](https://trace.ncbi.nlm.nih.gov/Traces/sra/sra.cgi?run=SRR6286470.554516.1&RID=4SHRHPC5016&display=reads) (99%) | TRINITY_GG_21764_c3_g1_i1 (99%) | *AF318949 (A. chinensis)*  (85%) | NA |
| *SPS2* | [GGAB01078977.1](https://www.ncbi.nlm.nih.gov/nuccore/GGAB01078977.1) | [324571](https://trace.ncbi.nlm.nih.gov/Traces/sra/sra.cgi?run=SRR6286470.324571.1&RID=43MVXADE014&display=reads) (99%) | TRINITY_GG_1633_c14_g1_i2 (99%) | *NC012017 (V. vinifera)*  (78%) | NA |
| *SPS3* | [GGAE01036876.1](https://www.ncbi.nlm.nih.gov/nuccore/GGAE01036876.1) | 1146340 (97%) | TRINITY_GG_12565_c121_g1_i1 (99%) | *ONI28760.1 (P. persica)*  (82%) | NA |

^1^The gene query match with the highest similarity corresponding transcript IDs from *V. myrtillus* transcriptomes dataset (Nguyen et al., 2018).

^2^ The gene query match with the highest similarity corresponding unigene IDs from *V. myrtillus* transcriptomes dataset (Samkumar et al., 2021)

^3^ The likelihood scores for cellular localization are shown in brackets in the range of 1 (NA-not applicable).

**Supplementary methods**

Plant-mSubP online tool (<http://bioinfo.usu.edu/Plant-mSubP/>)  by Sahu et al. (2019) was used to predict the subcellular localization of the INVs.

**References**

Sahu, S., Loaiza, C., & Kaundal, R. (2019). Plant-mSubP: a computational framework for the prediction of single- and multi-target protein subcellular localization using integrated machine-learning approaches. *AoB Plants*, 12(3), 68. doi: 10.1093/aobpla/plz068
